# Characterization of Mast Cells from Healthy and Varicose Human Saphenous Vein

**DOI:** 10.3390/biomedicines10051062

**Published:** 2022-05-03

**Authors:** Katrine T. Callesen, Sofia Mogren, Frida Berlin, Cecilia Andersson, Susanne Schmidt, Lotte Klitfod, Vanesa Esteban, Lars K. Poulsen, Bettina M. Jensen

**Affiliations:** 1 Laboratory of Medical Allergology, Copenhagen University Hospital, Gentofte, 2900 Hellerup, Denmark; lkpallgy@mail.dk (L.K.P.); bettinamjensen@hotmail.com (B.M.J.); 2 Respiratory Cell Biology, Department of Experimental Medical Science, Lund University, 22184 Lund, Sweden; sofia.mogren@med.lu.se (S.M.); frida.berlin@med.lu.se (F.B.); cecilia.andersson@med.lu.se (C.A.); 3 Department of Cardiothoracic Surgery, Rigshospitalet, 2100 Copenhagen, Denmark; susanne.schmidt@regionh.dk; 4 Department of Vascular Surgery, Gentofte Hospital, 2900 Hellerup, Denmark; klitfod@dadlnet.dk; 5 Department of Allergy and Immunology, IIS-Fundación Jiménez Díaz, 28040 Madrid, Spain; vesteban@fjd.es

**Keywords:** mast cells, vascular system, tryptase, histamine, MRGPRX2, PAF, C3a, C5a

## Abstract

Mast cells (MCs) are distributed in tissues throughout the body and are highly involved in many physiological and pathophysiological processes. The potential and involvement of different MC phenotypes are still not well understood. MCs are present in blood vessel walls, but their specific phenotypic features are unknown. We aimed at characterizing MCs from human saphenous veins for localization, mediator content, and receptor expression. This was done in MCs from both healthy and varicose human saphenous veins (hSV and vSV, respectively). For both vSV and hSV, we found that vein MCs are mainly present in the tunica adventitia (99% MCs in adventitia) and that the population consists of both MC_T_ and MC_TC_ phenotypes (vSV: 55% MC_T_, hSV: 64% MC_T_). The vein MCs contained high levels of histamine (vSV: 27 pg/MC, hSV: 55 pg/MC) and tryptase (vSV: 98 pg/MC, hSV: 111 pg/MC), indicating a strong potential for regulatory effects on blood vessels. The receptor expression of FcεRI, MRGPRX2, PTAFR, C3aR, and C5aR was found, even though the percentage of positive cells differed between vSV and hSV MCs. We conclude that vein MCs from the blood vessel wall have a high potential to affect the tissue around them.

## 1. Introduction

The mast cell (MC) is one of the major players in immunology and has a vast number of receptors that allow this cell type to interact with both the innate and adaptive parts of the immune system [1]. MCs are found in close proximity with the vasculature and though mainly known for their role in allergic reactions, urticaria, and the MC cancer mastocytosis, these cells are also involved in numerous diseases affecting the vascular system, e.g., arteriosclerosis, migraine, rheumatoid arthritis, and various cancers [1,2,3,4,5,6]. Once activated, MCs can release a wide range of mediators, including histamine and tryptase. However, due to high heterogeneity, the MC response varies between different tissues, both regarding what activates the MC, but also which mediators are released upon activation [1,2,7,8,9]. In addition, MCs have a large plasticity, giving them the capability to change their phenotype, e.g., upon environmental changes [10,11]. Therefore, in order to understand the potential and function of MCs within a tissue, the MCs from that specific tissue must be characterized, and due to their plasticity, the cells must be investigated as close to their natural surroundings as possible [2,7,10].

Human MCs have long been subdivided into two different phenotypes; MC_T_s that express the MC-specific protease tryptase and MC_TC_s that, besides tryptase, also express carboxypeptidase A3 and chymase [2,7,12]. Most research on human MCs has been conducted on skin and lung MCs, while other tissue MCs, such as from the heart, have been less frequently investigated. Lung MCs are mainly of the MC_T_ phenotype, while in both the skin and heart, it is the MC_TC_ phenotype that dominates [2,7,8]. The different MC populations also respond differently to some inflammatory stimuli including Complement 3a (C3a), Complement 5a (C5a), Substance P (SP), and platelet-activation factor (PAF). Of these, lung MCs only respond to PAF, while skin MCs respond to all except PAF. Heart MCs, despite also being of the MC_TC_ phenotype, respond to the same stimuli as skin MCs except for SP [2,9,13]. Their response to PAF is unknown. Lung, skin, and heart MCs all respond to anti-IgE [2,9,14].

MCs are known to be present in human blood vessel walls and, in large blood vessels, often in close proximity to the vasa vasorum [15,16,17]. Their phenotype and possible functions in the blood vessel wall however remain largely unknown. Many MC mediators have a fast and severe effect on the vascular system, including PAF and histamine that strongly affect vascular permeability and vasodilation [18,19]. Therefore, MCs located within the vascular wall might possess a great potential in the regulation of blood vessel responses in pathophysiological reactions. To further investigate this, a characterization of vascular MCs is needed. Therefore, the aim of this study was to characterize the MCs from human saphenous veins in relation to their localization, mediator content, and receptor expression. This investigation was done in MCs from healthy saphenous veins (hSV) that were left over from cardiac arterial bypass grafts. To investigate the potential useability of more readily available tissues, MCs from varicose saphenous vein (vSV) were also included and compared to the results from MCs from hSV.

We found that both MC_T_ and MC_TC_ phenotypes are present in human saphenous veins and that they contain high levels of both histamine and tryptase compared to those found in MCs from other tissues. These vein MCs (vMCs) express the receptors FcεRI, MRGPRX2, PTAFR, C3aR, and C5aR. Overall, the MCs from hSV and vSV are largely similar. Based on these findings, vMCs appear to possess a different MC phenotype than that previously reported in other tissues and have a high potential to cause severe effects on blood vessels upon activation.

## 2. Materials and Methods

### 2.1. Material Origin and Initial Processing

#### 2.1.1. Human Saphenous Veins

Two sources of human vena saphena were included in this study. Varicose saphenous veins (vSV) were acquired from the Department of Vascular Surgery at Gentofte Hospital, Denmark after surgical removal due to varicosity (ethical approval H-4-2013-082). Healthy saphenous veins (hSV) were acquired from the Department of Cardiothoracic Surgery at Rigshospitalet, Denmark after surgical removal for cardiac arterial bypass graft where small pieces of vein were left over (ethical approval H-19085670). This study was conducted from 2015 to 2019, and informed consent was obtained from all patients. For both sources of vena saphena, the collection of samples took place as part of an already required surgery (either for varicose vein removal or for use in cardiac arterial bypass grafts). No extra pieces of vein were subtracted due to the current study and only leftover material from the already planned surgeries were used. Therefore, no clinical implications were present for any of the patients.

After removal, the vein (both vSV and hSV) was transferred to the laboratory in PBS and used for either tissue embedding or the enzymatic extraction of cells.

#### 2.1.2. LAD2

LAD2 cells were cultured in LAD2 medium (StemPro-34 SFM (Gibco, Waltham, MA, USA) and StemPro-34 SFM supplement (Gibco) with 100 U/mL penicillin (Sigma-Aldrich, St. Louis, MO, USA), 100 µg/mL streptavidin (Sigma-Aldrich), 2 mM L-glutamine (Sigma-Aldrich), and 100 ng/mL rhSCF (Preprotech, Rocky Hill, NJ, USA) in a concentration of 300,000 cells/mL. When used, 1,000,000 live cells were taken, centrifuged, and prepared for lysis of cells by resuspension in 1 mL demineralized H_2_O (ddH_2_O).

#### 2.1.3. Peripheral Blood Derived Mast Cells

Peripheral blood derived mast cells (PBdMCs) were matured from CD34+ stem cells as previously described [20,21]. In brief, peripheral blood mononuclear cells were purified from the buffy coats of anonymous donors from the Danish National Blood Bank using lymphoprep gradient separation. Buffy coat blood was mixed 1:1 with PBS and underlaid with lymphoprep (Alere Technologies AS, Oslo, Norway) in a volume corresponding to 1/3 of the blood/PBS volume. After centrifugation at 400× *g*, 30 min, RT, the white blood cell interphase layer was harvested and mixed with 150 mL PBS. This was centrifuged 300× *g*, 10 min, RT and the pellet resuspended in 150 mL PBS for further centrifugation at 200× *g*, 10 min, RT. The pellet was then resuspended in 50 mL MACS buffer (PBS, 0.5% HSA (CLS Behring, King of Prussia, PA, USA), and 2 mM EDTA (hospital pharmacy)), and the total cell number and viability were determined. CD34+ purification was conducted using a CD34 MicroBead Kit (MACS Miltenyi Biotec, Bergisch Gladbach, Germany) according to the manufacturer’s instructions.

After purification, the cells were cultured in PBdMC medium (LAD2 medium and 100 ng/mL rhIL-6 (Preprotech)) with the addition of 30 ng/mL of rhIL-3 (Preprotech) during the first week. Fresh PBdMC medium was added to the cultures weekly for the first three weeks. After this, the medium was replenished completely once a week. After culturing for 7–8 weeks, the mature PBdMCs were split into two; 100,000 total cells were used for cytospin and subsequent toluidine blue staining to check the purity of the cultures (>90% in all). The remaining cells were resuspended in 200,000 cells/mL of ddH_2_O for the subsequent lysis of cells.

### 2.2. Tissue Embedding

#### Paraffin Preparation

Vein pieces of 0.5–1 cm were immediately transferred to 4% formaldehyde (Histolab, Västra Frölunda, Sweden) and incubated for 24 h at RT, followed by dehydration and paraffin embedding. Sections were cut at 4 µm thickness and used for immunohistochemistry.

### 2.3. Staining Methods

#### 2.3.1. Toluidine Blue Staining

Glass slides with cell cytospin were fixated by incubating with MOTA’s fixative (50 mL ddH_2_O, 40 mg/mL lead acetate (Fisher Scientific, Waltham, MA, USA), 2–4 mL glacial acetic acid (Merck, Kenilworth, NJ, USA), and 50 mL 96% EtOH (Sigma-Aldrich)) for 15 min, washed with ddH_2_O and incubated for 30 min with acidic toluidine blue (30 mL 96% EtOH and 5 mg/mL toluidine blue (Sigma-Aldrich) and 70 mL ddH_2_O, pH adjusted to <1 with HCl). After incubation, the glass slides were washed with ddH_2_O and coverslipped with Permount Mounting Medium (Permount).

#### 2.3.2. Immunohistochemistry, Double HRP Staining

MC_T_/MC_TC_ identification was made using the Dako EnVision G|2 Doublestain System kit (Dako) as described by the manufacturer, with antibodies against tryptase and chymase (detailed in Table 1). The double stained tissue sections were scanned through a 20× microscope lens by an automated digital slide-scanning robot (Scanscope CS, Aperio, Vista, CA, USA). MCTC and MCT phenotypes were manually quantified in a blinded manner and related to the tissue areas that were calculated using ImageScope (v10.0.36.1805, Aperio).

#### 2.3.3. Immunohistochemistry, Fluorescence

Following antigen retrieval in a target retrieval solution of low pH (Dako PT Link, Dako, Glostrup, Denmark)) for 1 h and the blocking of unspecific binding for 20 min (Dako protein block, serum-free (Dako)), double staining was conducted by staining the marker of interest (against FcεRI, PTAFR, C3aR, C5aR, or MRGPRX2) followed by an appropriate secondary antibody (the full overview of primary and secondary antibodies can be found in Table 1). All antibodies were diluted in Dako antibody diluent (Dako). After this, direct staining of tryptase was carried out using the Zenon™ Alexa Fluor™ 488 Mouse IgG1 Labeling Kit (Invitrogen, Carlsbad, CA, USA) with monoclonal mouse anti-tryptase (Sigma-Aldrich, #MAB1222, 1:100 dilution). For nuclei staining, the slides were mounted with ProLong Gold Antifade reagent with DAPI (Invitrogen). After double immunofluorescence staining, the filter setting was adjusted to reveal the tryptase-positive mast cells at 488 nm. By alternating the filter settings, each tryptase-positive cell was examined for the presence of receptor expression (555 nm). The percentage of receptor-expressing mast cells was calculated by dividing the number of receptor+ MCs with the total number of tryptase+ MCs multiplied by 100.

### 2.4. Enzymatic Extraction

Vein pieces were cut into 4 cm pieces and blood washed away. After this, the vein was cut into pulp, weighed, and incubated for 1 h at 37 °C, rotating in an enzymatic mix of 2540 U/g tissue of collagenase I (Worthington, Columbus, OH, USA) and 11,700 U/g tissue of collagenase II (Worthington) in 9.25 mL/g tissue of tissue medium (DMEM/F-12 (Gibco) and 100 U/mL penicillin and 100 µg/mL streptomycin). After incubation, 925 µL/g tissue of fetal bovine serum (Sigma-Aldrich) was added, the tissue flushed through a 14G needle 20 times, and the cells isolated by passing through a 70 µm cell strainer (Falcon, Mersin, Turkey). After centrifugation, the cells were resuspended in 750 µL/g tissue of PBS. A total of 100 µL of the suspension was used for cytospin, while the remaining volume was centrifuged and resuspended in 0.5–1 mL ddH_2_O to use for the lysis of cells.

### 2.5. Cytospin and Quantification

A total volume of 100 µL cells in PBS was added to an EZ single cytofunnel (Thermo Scientific, Waltham, MA, USA) with a superfrost plus glass slide (Menzel Gläser, Bad Wildungen, Germany) and centrifuged 600 rpm for 5 min. After this, the glass slide was stained with toluidine blue as described in the previous section.

For enzymatically extracted cells from the vein, the stained cytospin was used to quantify the number of MCs in the cell suspension by subsequently counting the number of MCs via microscope. This number was then used to assess the number of MCs in the remaining suspension of cells, which was used for each lysate in the following equation:

$$Estimated\;MCs\;in\;lysate=MCs\;counted\;on\;cytospin\times \frac{\left(Total\;vol.\;of\;cells-vol.\;used\;for\;cytospin\right)}{Vol.\;used\;for\;cytospin}$$

### 2.6. Lysing of Cells

Cells were lysed by repeated freeze/thaw cycles and mechanical disruption by passing cells 20× through a 26 G needle. After 3 cycles, the suspension was investigated by microscope for intact cells, and, if needed, the freeze/thaw cycle was repeated. After lysing, the suspension was centrifuged at 500× *g*, 10 min, RT, and the supernatant was transferred for use in histamine and tryptase measurements.

### 2.7. Histamine and Tryptase Measurements

Suspensions were diluted in Pipes buffer (Reflab, Copenhagen, Denmark) for histamine and in tissue medium for tryptase.

Histamine was detected by incubating 3 × 25 µL diluted suspension for 30 min, 37 °C on a glass fiber-coated microtiter plate (Reflab, Zürich, Switzerland) that had been reconstituted with 25 µL Pipes buffer. After incubation, the plates were washed with ddH_2_O. A total of 175 µL 0.4% sodium dodecyl sulfate (Reflab) in ddH_2_O was added to each well, and the plate was incubated for 10 min, 37 °C followed by washing with ddH_2_O. The washed plates were incubated for 10 min with 0.1 mg/mL o-phthaldialdehyde (Reflab) in 50 mM sodium hydroxide (hospital pharmacy). The reaction was stopped using 0.59% perchloric acid (hospital pharmacy), and the plate was read on the Histareader system according to the manufacturer’s instructions. 

Tryptase was measured using the Phadia ImmunoCAP (Thermo Fisher Diagnostics, Milton Keynes, UK). The limit of detection was 1 ng/mL.

The measured levels were combined with the MC estimation made using the cytospin (or for PBdMCs and LAD2, the already known MC concentration) and used to assess the pg histamine or tryptase per MC via the following equation:

$$pg\;histamine\;or\;tryptase/MC=\frac{Measured\;histamine\;or\;tryptase\;\left(ng/ml\right)}{Estimated\;MCs\;in\;lysate}\times 1000\;pg/ng$$

### 2.8. Statistical Analysis

Because of the low number of replicates, a normal distribution could not be assumed, and therefore, where applicable, non-parametric tests were used. Mann–Whitney tests were used to compare between groups, and correlations were assessed with the Spearman correlation. Data are expressed as median values with the interquartile range. Analysis was performed using Graphpad Prism 8.0.2.

## 3. Results

### 3.1. Histology and MC_T_/MC_TC_ Distribution

**Figure 1:**
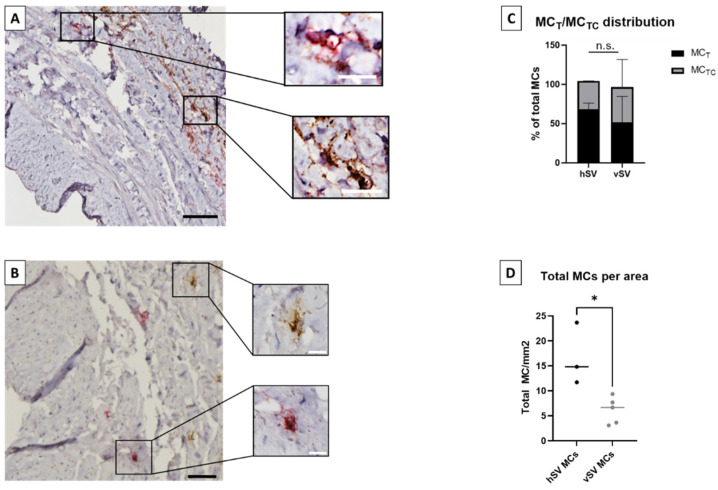
Localization and distribution of mast cells (MCs) in vein. (**A**,**B**): Illustration of MC localization in human saphenous vein: several 4 µm sections of healthy saphenous vein (hSV, (**A**)) or varicose saphenous vein (vSV, (**B**)) were stained with double horseradish peroxidase staining. Red staining: MC_T_. Brown staining: MC_TC_. Black bar: 100 µm, white bar: 10 µm. (**C**): Distribution of MC_T_ and MC_TC_ in both hSV (*n* = 3) and vSV (*n* = 5). (**D**): Overview of total MC counted per mm^2^ in hSV (*n* = 3) and vSV (*n* = 5). * *p* < 0.05, n.s.: non-significant *p*-value.

The vMCs were almost exclusively detected in tunica adventitia (99% in adventitia for vSV (*n* = 5) and 100% for hSV (*n* = 3), Figure 1A,B), often in close proximity to vasa vasorum.

Both the MC_T_ and MC_TC_ phenotypes were present in the veins (MC_T_ in vSV (median/IQR): 55%/20–82%, in hSV: 64%/64–77%, Figure 1C). No differences were observed between vSV and hSV in the location of the vMCs nor in the MC_T_/MC_TC_ distribution (Figure 1C) However, a higher number of MCs per area was observed in hSV compared to vSV (Figure 1D).

### 3.2. Histamine and Tryptase Content

**Figure 2:**
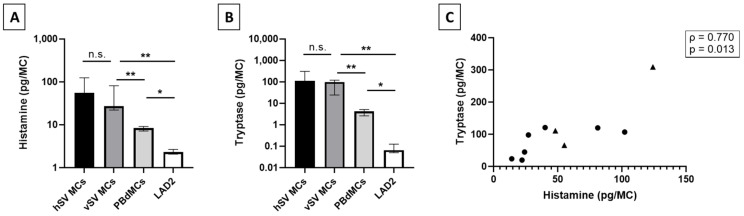
Histamine and tryptase content in mast cells (MCs) from vein or in vitro cultures. MCs from healthy saphenous vein (hSV MCs, *n* = 3), from varicose saphenous vein (vSV MCs, *n* = 7), PBdMCs (*n* = 4), and LAD2 (*n* = 4) were lysed by repeated cycles of freeze-thawing and mechanical disruption. The lysed solutions were used to measure the pg/MC of histamine (**A**) and tryptase (**B**). (**C**): Correlation of histamine and tryptase in MCs from hSV (triangle, *n* = 3) and vSV (circles, *n* = 7) saphenous vein was plotted and correlation tested using Spearman correlation. * *p* < 0.05, ** *p* < 0.01, n.s.: non-significant *p*-value.

**Figure A1:**
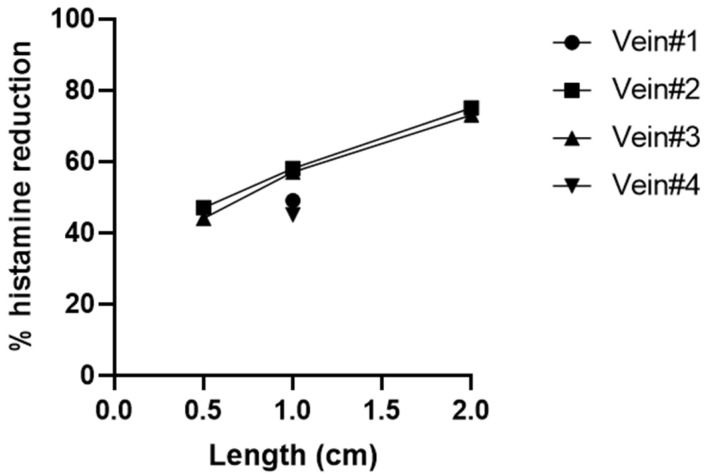
Histamine removal of unstimulated vein. Varicose saphenous vein of 0.5 cm (*n* = 2), 1 cm (*n* = 4) or 2 cm (*n* = 2) pieces were incubated 1 h with 200 ng/mL histamine in supernatant. % histamine reduction was calculated by comparing measured histamine after incubation with histamine levels of control supernatant (tube ran in parallel without added vein piece). #: Number of vein tested.

In the attempt to measure histamine in MCs from saphenous veins, we discovered that the vein itself demonstrated a high ability to remove histamine, as a 1 cm vein incubated with 200 ng/mL histamine for 1 h removed 52% of the histamine (*n* = 4, Figure A1). Therefore, we enzymatically extracted the cells before measuring their histamine and tryptase contents to ensure an optimal readout. Both vSV and hSV MCs expressed a high histamine content (vSV (median/IQR): 27 pg/22–81 pg, hSV: 55 pg/48–124 pg (Figure 2A)) and tryptase content (vSV: 98 pg/24–120 pg, hSV: 111 pg/66–309 pg (Figure 2B)). The levels of histamine and tryptase were significantly correlated (*n* = 10, ρ = 0.770, *p* = 0.013, Spearman correlation, Figure 2C), and no difference was detected between vSV and hSV for either histamine or tryptase content.

### 3.3. Comparison to Mast Cell In Vitro Models

As MCs are challenging to isolate from tissue and culture in vitro, MC models are often used. These include the cancer cell line LAD2 and CD34+ peripheral blood derived MCs (PBdMCs). Therefore, these two in vitro cultured MC models were also investigated for histamine and tryptase content (Figure 2A,B). We found very low levels of histamine and tryptase in LAD2 (histamine (median/IQR): 2.3 pg/2.1–2.7 pg, tryptase: 0.07 pg/0.05–0.13 pg). The content was significantly higher for PBdMCs (histamine: 8.3 pg/7.2–9.1 pg, tryptase: 4.2 pg/2.6–5.1 pg, *p* = 0.029). Both LAD2 and PBdMCs had a significantly lower histamine and tryptase level compared to vMCs (*p* = 0.006 for all).

### 3.4. Receptor Expression

**Figure 3:**
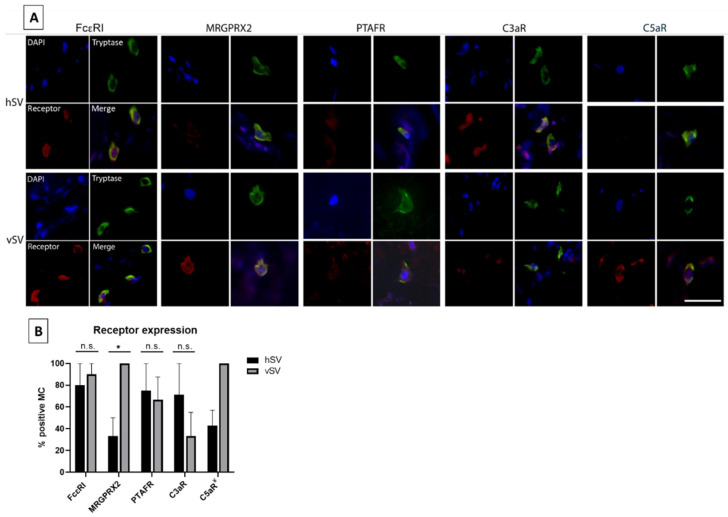
Receptor expression in mast cells (MCs) from vein. (**A**): Several 4 µm paraffin sections of varicose saphenous vein (vSV, *n* = 5) or healthy saphenous vein (hSV, *n* = 3 ^¥^) were stained for tryptase to identify MCs and co-stained with either FcεRI, MRGPRX2, PTAFR, C3aR, or C5aR. Scale bar: 20 µm. (**B**): Comparison of receptor expression between hSV and vSV. For each vein, the number of MCs expressing the specific receptor was counted and compared to the total number of MCs present (based on tryptase expression). * *p* < 0.05, n.s.: non-significant *p*-value. ¥: *n* = 2 for C5aR expression due to lack of MC detection.

Expression levels of FcεRI, MRGPRX2, PTAFR, C3aR, and C5aR were observed in vMCs from both hSV and vSV (Figure 3A). Differences between hSV and vSV were observed, especially for MRGPRX2, whereby most vMCs from vSV were positive (median: 100%, IQR: 87.5–100%) compared to vMCs from hSV (median: 33.3%, IQR: 0–50%, *p* = 0.018). The same trend was observed for C5aR (median: 100%, IQR: 35–100% for vSV), although only two datapoints were obtained for hSV (28.6% and 57.1% respectively). An opposite tendency was found for C3aR, with fewer vMCs expressing the receptor in vSV (median: 33.3%, IQR: 31–55%) compared to vMCs from hSV (median: 71.4%, IQR: 50–100%, *p* = 0.071). No differences were found in the expression of FcεRI and PTAFR (Figure 3B).

## 4. Discussion

The vascular system is one of the fastest signaling routes around the body. From mouse studies it has been shown that MCs in close contact with the blood vessels can be greatly affected by components from the blood [22,23]. Furthermore, many of the mediators released from MCs (including histamine and PAF) can have a great impact on vascular permeability and overall function [3,23]. MCs from the human vascular system are, however, not well characterized and their specific potential remains to be uncovered.

Therefore, we investigated vMCs from human saphenous veins both on a histological basis, for mediator content, and receptor expression. This was investigated both in healthy veins that were left over from cardiac arterial bypass graft (hSV) and in veins that were surgically removed due to varicosity (vSV). vSV were included since human varicose veins are easier to acquire for experimental use, and thus, future investigations could be furthered by the possibility of testing MCs from vSV rather than from hSV.

In vena saphena, MCs have previously been reported to be present in tunica adventitia and often close to vasa vasorum [15,16,17], which corresponded to our findings. The vMC population was found to consist of both MC_T_ and MC_TC_, with a tendency of more MC_T_ than MC_TC_ (Figure 1C). Andersson et al. [8] have previously investigated MCs from pulmonary vessels in which they found a similar MC_T_/MC_TC_ distribution.

Previous reports on mediator content in human MCs from lung, skin, and heart describe these MCs as having a similar level of histamine with the highest being detected in MCs from skin (1–7 pg/MC) [9,12,14,24,25]. Interestingly, we detected a level of histamine close to 10 times higher in the vMCs compared to other tissues, whereas the levels found in LAD2 and PBdMCs were comparable to previous reports [14,21,26] (Figure 2A). Tryptase has been found in varying levels between lung, skin, and heart MCs [9,12,14]; in this case, we detected a much higher level in the vMCs (Figure 2B). The surprising finding of high levels of both histamine and tryptase in vMCs could indicate that vMCs are very potent and that they, upon activation, have the potential to strongly affect their surroundings. This could potentially cause a huge impact on blood vessel permeability in vivo which can have severe consequences such as hypoxia and a drop in blood pressure [19]. As has also been shown in skin MCs [25], we found a correlation between the total tryptase and histamine in vMCs (Figure 2C). The lower levels of both histamine and tryptase detected in both LAD2 and PBdMCs indicate that these two MC models are not optimal surrogates for studies on mediator release from vMCs, at least in relation to histamine and tryptase content. 

The receptor expression of MCs has been found to vary in different tissues throughout the body. We therefore selected five receptors to investigate in vMCs. FcεRI, as well as four receptors (C3aR, C5aR, PTAFR, and MRGPRX2) in which receptor expression (or the response to stimuli of these receptors) has been found to vary between MC phenotypes [2,3,9,13] were investigated. We have shown that almost all vMCs express FcεRI (Figure 3B). In contrast, Andersen et al. [8] only detected a low number of pulmonary vessel MCs expressing FcεRI, indicating that there might be some phenotypical differences between MCs throughout the vascular system. We also found the expression of the other four receptors in vMCs (Figure 3A,B). Since the mediators activating these receptors are known to be released and involved in various severe reactions, including anaphylaxis, this—combined with the high level of histamine found in vMCs—gives these MCs the potential to have a high impact on the vascular system in some pathological situations.

Our results showed that the vSVs can serve well as study objects in many aspects, as no difference was found between vSV and hSV in MC_T_/MC_TC_ distribution (Figure 1C), levels of tryptase, histamine content (Figure 2A,B), or FcεRI and PTAFR expression (Figure 3B). However, a few differences were found. The vSVs have previously been shown to display an increased number of MCs [15,16], possibly due to a change in the microenvironment which might affect the MC phenotype. Interestingly, we found the opposite trend (Figure 1D). The reasons for this are currently unknown. A difference was also observed in the number of MCs positive for MRGPRX2 (Figure 3B), with MCs from vSV having the highest number. Whether this reflects a phenotypical change is uncertain but nonetheless, some MCs from both vSV and hSV express FcεRI, PTAFR, MRGPRX2, C3aR, and C5aR (Figure 3A,B). One could speculate that despite having fewer MCs, the degranulation magnitude might be higher in vSVs due to the increased receptor number resulting in a stronger response towards neuropeptides in MCs from vSV compared to hSV.

In conclusion, MCs from veins consist both of MC_T_ and MC_TC_ phenotypes and express receptors for IgE, SP, PAF, C3a, and C5a. Most surprisingly, these MCs contain a very high amount of both histamine and tryptase compared to what has been reported previously in different tissue MCs. Thus, vMCs possess the capability to affect the vasculature and drive a systemic reaction. Overall, MCs from vSV resemble the phenotype of those found in hSV and thus could be utilized in future studies of the function and responses of vMCs.

## Figures and Tables

**Table 1:** Primary and secondary antibodies used for immunohistochemistry.

Antibody *	Dilution	Cat.no	Manufacturer
Mouse anti-tryptase (monoclonal)	1:100/1:1000	MAB1222	Chemicon
Mouse anti-chymase (monoclonal)	1:100	MA5-11717	Thermo Fisher Sci
Mouse anti-C3aR (monoclonal)	1:50	sc-133172	Santa Cruz Biotechnology
Mouse anti-FcεRI (monoclonal)	1:50	LS-B4054	LSBio
Rabbit anti-PTAFR (polyclonal)	1:50	HPA027543	Sigma-Aldrich
Rabbit anti-MRGPRX2 (polyclonal)	1:400	HPA055220	Sigma-Aldrich
Rabbit anti-C5AR1 (polyclonal)	1:50	HPA014520	Sigma-Aldrich
Alexa Fluor 555-conjugated goat anti-rabbit IgG	1:200	A21428	Invitrogen
Alexa Fluor 555-conjugated goat anti-mouse IgG	1:200	A21422	Invitrogen

* For all immunohistochemical procedures, markers, and tissues, staining was absent in sections using isotype-matched control antibodies (Dako) that were used instead of, and in the same concentration as, the primary antibody.

## Data Availability

Research data are not shared.
